# An uncommon presentation of Kikuchi Fujimoto disease: a case report with literature review

**DOI:** 10.1186/s13104-015-1460-x

**Published:** 2015-09-26

**Authors:** Sabin Ranabhat, Mamta Tiwari, Jiwan Kshetri, Sushna Maharjan, Bidur Prasad Osti

**Affiliations:** Department of Pathology, Chitwan Medical College (CMC) Teaching Hospital, Bharatpur-10, Chitwan, Nepal

**Keywords:** Kikuchi, Fujimoto, Lymphadenitis, Thrombocytopenia, Nepal

## Abstract

**Background:**

Kikuchi–Fujimoto disease is so named because Kikuchi and Fujimoto were the first scientists to describe it in Japan in 1972. Although the disease has been reported from all over the world and more so from Asia, it is rare. To date only eight cases have been reported from Nepal. Cervical lymphadenopathy, fever and raised Erythrocyte Sedimentation Rate are usual presenting features of this disease. We describe a case which presented with thrombocytopenia and axillary lymphadenopathy in addition to the usual features. Out of the total eight cases that have been reported from Nepal so far, no patients had thrombocytopenia and only one patient had axillary lymphadenopathy.

**Case presentation:**

A 24-year-old Nepali female presented with a 3-week history of low-grade fever, headache, and painful, discrete, unilateral left-sided cervical and axillary lymphadenopathy. Among the multitude of tests that were carried out, Erythrocyte Sedimentation Rate was raised and there was thrombocytopenia while other tests were normal. Painful lymphadenopathy pointed to bacterial lymphadenitis while chronic low-grade fever suggested tuberculosis. A cervical lymph node was excised for histopathological examination to reach an accurate diagnosis. On the basis of pathognomonic features viz., paracortical foci composed of various types of histiocytes including crescentic type in the background of abundant apoptotic karyorrhectic debris, a diagnosis of Kikuchi–Fujimoto disease was made. On follow-up evaluation after 6 weeks, the patient had no systemic symptoms, enlarged lymph nodes had regressed in size significantly, and Erythrocyte Sedimentation Rate and platelet count had become normal.

**Conclusion:**

Kikuchi–Fujimoto disease should be kept in the differential diagnosis of lymphadenopathy in young patients, female or male even in tuberculosis-endemic countries and even in patients who have unusual features; for example thrombocytopenia and involvement of axillary lymph nodes in addition to cervical lymph nodes as in this case.

## Background

Kikuchi–Fujimoto disease (KFD), also known as histiocytic necrotizing lymphadenitis, was first described independently by two Japanese scientists, Dr. Kikuchi and Dr. Fujimoto in the same year, 1972. The disease process subsides within 1–4 months without specific treatment [1]. The exact cause of the disease is not known, although the viral etiology is suspected [2]. KFD is a rare disease having world-wide distribution, but it is more common in Asia. The disease is more common in young females than males. Most of the patients present with unilateral, painful or painless cervical lymphadenopathy and fever. Axillary and inguinal lymph nodes are less frequently involved. Diagnosis of KFD is achieved by histopathological examination of affected lymph nodes [1]. There are no laboratory findings specific to KFD. Anemia, elevation of ESR and mild leukopenia may be found in some patients [3]. Thrombocytopenia is a rare finding [4].

Clinical presentation of low-grade fever with lymphadenopathy can cause confusion with tuberculosis, toxoplasmosis and infectious mononucleosis [5]. Non-Hodgkin’s lymphoma comes in the list of differential diagnoses, especially when there are atypical immunoblasts [6].

To date only eight cases have been reported from Nepal [7–9]. In the patient of this case report, two uncommon findings were present along with other usual findings viz., thrombocytopenia and combined cervical plus axillary lymphadenopathy.

## Case presentation

Present case deals with a 24-year-old Nepali female patient who presented with a 3-week history of low-grade fever, fatigue, headache, and lymphadenopathy. She reported no weight loss, night sweats and anorexia. The patient did not have a history of tuberculosis and other significant illnesses in the past. The patient complained of painful unilateral left-sided cervical and axillary lymphadenopathy. Enlarged lymph nodes were found to be tender and discrete. Largest lymph node was in the cervical region which measured 3 × 3 cm.

Laboratory investigations for complete blood count, Erythrocyte Sedimentation Rate (ESR), liver function tests, renal function tests, anti-nuclear antibody and rheumatoid arthritis factor were performed. ESR as measured by Westergren method was 64 mm/h (normal range: <20 mm/h in females). Platelet count was 120,000/µl (normal range: 150,000–450,000/µl). Other test findings were within normal limits, including hemoglobin and total and differential leukocyte count.

There was a dilemma in clinical diagnosis because lymphadenopathy was painful, but the patient had raised ESR and low-grade fever. Painful lymphadenopathy pointed to reactive (bacterial) lymphadenitis while increased ESR and low-grade fever pointed to tuberculosis. Therefore empirical treatment was started with a 1-week’s course of broad-spectrum antibiotic. But the patient did not show any improvement and therefore cervical lymph node was excised for histopathological examination.

**Fig. 1:**
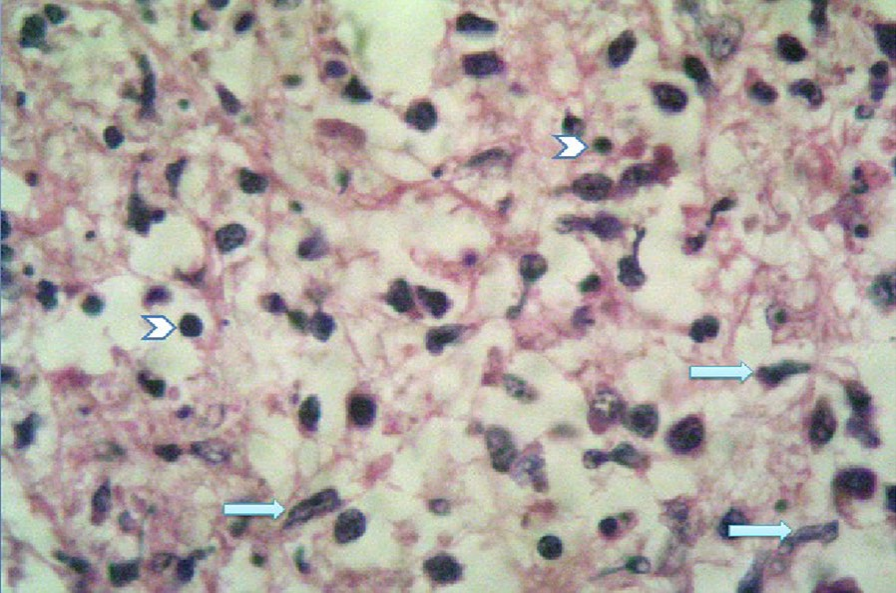
High power view showing apoptotic bodies (*arrow heads*) and histiocytes (*arrows*). H&E ×40 objective

**Fig. 2:**
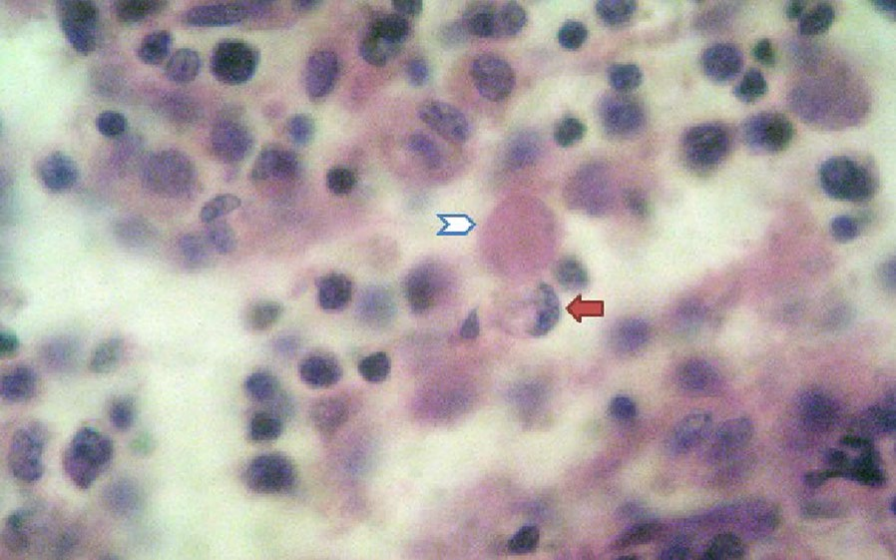
High power view showing ghost cell (*arrow head*) and crescentic histiocyte (*arrow*). H&E ×40 objective

Low power microscopic examination of section prepared from affected lymph node showed discrete necrotic area. Pathognomonic features of KFD were seen on higher power microscopic examination viz., paracortical foci composed of small to large lymphocytes, plasmacytoid monocytes and various types of histiocytes (Fig. 1) including crescentic type (Fig. 2) in the background of apoptotic bodies and abundant karyorrhectic debris (Fig. 1). Ghost cell represented complete karyorrhexis (Fig. 2). Neutrophils, eosinophils and plasma cells were conspicuous by their absence. Lymphocytes did not show atypia. Mitotic figures were not present. There were no granulomas. Microscopic features of lupus lymphadenopathy viz., follicular hyperplasia with interfollicular immunoblasts and Reed-Sternberg-like giant cells and paracortical necrosis with hematoxylin bodies were absent. On the basis of these histopathological findings, a diagnosis of Kikuchi–Fujimoto disease (histiocytic necrotizing lymphadenitis) was made. Immunohistochemical analysis was not done on the sections because of confirmed diagnosis on the basis of light microscopic examination of Hematoxylin and Eosin stained sections.

### Follow-up visit

During the evaluation after 6 weeks, the patient reported no systemic symptoms and her lymph nodes had regressed in size significantly and ESR and platelet count were restored to normal range, without any treatment.

## Discussion

### Epidemiology

Although uncommon, KFD has been described from all over the world [10]. Most cases have been reported from Asia [1]. However, only eight cases have been reported from Nepal, a country in Asia.

### Lymph nodes affected

Cervical lymph nodes have been found to be affected in nearly all the patients in previous studies. Cervical lymph nodes were affected in seven and axillary lymph nodes in only one out of the eight cases reported from Nepal [7–9]. In the 11 cases reported from India, involvement of cervical lymph nodes was 100 %. Only one patient had generalized lymphadenopathy [5, 11–16]. In a retrospective study of 195 patients by Cheng et al., only 2.6 % of patients had axillary lymphadenopathy [17]. These studies indicate that the involvement of axillary lymph nodes is not common in KFD.

### Laboratory findings

Although no laboratory finding is specific for KFD, leukopenia and raised ESR may be present. Leukopenia was present in 31.3 % patients in a study of 96 cases by Kwon et al. [18]. Raised ESR was found in all patients in a study of six cases by Adhikari et al. [7]. Among the six patients reported from India, raised ESR was observed in five cases, and leukopenia and leukocytosis were found in one patient each [5, 11, 13–16]. Thrombocytopenia is rare. It was not present in any of the eight cases reported previously from Nepal [7–9]. In the study by Cheng et al., leukopenia, leukocytosis and thrombocytopenia were present in 18.9, 1.4 and 5.4 % of patients, respectively [17].

### Differential diagnoses

Because most of the patients of KFD present with localized lymphadenopathy and low-grade fever, it mimics tuberculosis clinically; this is particularly true in countries where tuberculosis is endemic, for example Nepal and other South Asian countries [1, 19]. Tuberculosis is so common in Nepal that it is the number one provisional diagnosis for any longstanding nontender lymphadenopathy. In this case report, although lymphadenopathy was longstanding it was painful too. Therefore, bacterial lymphadenitis was number one provisional clinical diagnosis followed by tuberculosis. This case gives a lesson to keep KFD as one of the potential diagnoses even in countries where tuberculosis is endemic. There are some more diseases which come in the differential diagnosis of KFD viz., non-Hodgkin lymphoma, systemic lupus erythematosus, infectious mononucleosis, toxoplasmosis and cat scratch disease. Although fine needle aspiration cytology (FNAC) is less invasive and cheaper, and turnaround time for diagnosis is shorter, its accuracy is only 56 %. Nevertheless, in the presence of clear-cut clinical findings, a diagnosis of KFD can be made by FNAC if unequivocal cytological features are present. But when unusual clinical features are present as in this case, histopathological evaluation of affected lymph nodes is the preferred diagnostic method [1, 10]. KFD has been classified histopathologically into three evolving phases: proliferative, necrotizing and xanthomatous. The first phase is characterized by the presence of histiocytes and plasmacytoid monocytes in the background of apoptotic karyorrhectic debris. Our case has histopathological features of this phase. In necrotizing phase, coagulative necrosis is also present and in xanthomatous phase, foamy histiocytes predominate [10].

## Conclusion

Kikuchi–Fujimoto disease should be kept in the differential diagnosis of lymphadenopathy in young patients, female or male even in tuberculosis-endemic countries and even in patients who have unusual features; for example thrombocytopenia and involvement of axillary lymph nodes in addition to cervical lymph nodes as in this case.

## Consent

Written informed consent was obtained from the patient for publication of this case report and the accompanying images. A copy of the written consent is available for review by the Editor-in Chief of this journal.

## Abbreviations

KFD: Kikuchi–Fujimoto disease
ESR: Erythrocyte Sedimentation Rate
H&E: hematoxylin and eosin
FNAC: fine needle aspiration cytology
